# Flexible Pressure Sensors and Machine Learning Algorithms for Human Walking Phase Monitoring

**DOI:** 10.3390/mi14071411

**Published:** 2023-07-13

**Authors:** Thanh-Hai Nguyen, Ba-Viet Ngo, Thanh-Nghia Nguyen, Chi Cuong Vu

**Affiliations:** Faculty of Electrical and Electronics Engineering, Ho Chi Minh City University of Technology and Education, 01 Vo Van Ngan Street, Linh Chieu Ward, Ho Chi Minh City 700000, Vietnam; nthai@hcmute.edu.vn (T.-H.N.); vietnb@hcmute.edu.vn (B.-V.N.); nghiant@hcmute.edu.vn (T.-N.N.)

**Keywords:** soft capacitive pressure sensors, healthcare, connectivity, random forest, flexible embedded system

## Abstract

Soft sensors are attracting much attention from researchers worldwide due to their versatility in practical projects. There are already many applications of soft sensors in aspects of life, consisting of human-robot interfaces, flexible electronics, medical monitoring, and healthcare. However, most of these studies have focused on a specific area, such as fabrication, data analysis, or experimentation. This approach can lead to challenges regarding the reliability, accuracy, or connectivity of the components. Therefore, there is a pressing need to consider the sensor’s placement in an overall system and find ways to maximize the efficiency of such flexible sensors. This paper proposes a fabrication method for soft capacitive pressure sensors with spacer fabric, conductive inks, and encapsulation glue. The sensor exhibits a good sensitivity of 0.04 kPa^−1^, a fast recovery time of 7 milliseconds, and stability of 10,000 cycles. We also evaluate how to connect the sensor to other traditional sensors or hardware components. Some machine learning models are applied to these built-in soft sensors. As expected, the embedded wearables achieve a high accuracy of 96% when recognizing human walking phases.

## 1. Introduction

Nowadays, intelligent devices use a large number of different sensors. Their applications are found in manufacturing, environmental protection, biotechnology, medical diagnostics, marine exploration, and space development. Large amounts of signals are collected in each of the above unique environments. New sensor technology has evolved along the following trends: developing novel materials and processes, realizing sensor integration and intelligence, and realizing the hardware system.

Wearable technology refers to electronic devices worn on the user’s body. These devices exist in various forms, including accessories, jewelry, clothing, and medical devices. The complexity of wearable devices varies. The most complex examples include Google Glass, artificial intelligence (AI) hearing aids, Microsoft’s HoloLens, or holographic computers—virtual reality (VR) headsets. Less sophisticated products are disposable skin patches with sensors that transmit patient data to monitoring equipment in a healthcare facility. Wearable devices contain built-in sensors that allow monitoring of body signals, location tracking, or biometric recognition. Most of these devices are attached to clothing/footwear and operate with or without direct contact with the human body. Smart cards and smartphones are portable and track users’ movements. Some wearable devices use remote sensors and accelerometers to recognize human motion and speed. Others use optical or chemical sensors to measure glucose, blood oxygen, and heart rates. One thing these devices have in common is that they all operate in real-time.

Among the new types of sensors, flexible soft sensors emerge as the ideal technology for the future of wearables. Flexible soft sensors are a general concept for sensors that can be applied to a variety of soft surfaces with different irregular shapes or spaces, such as human skin and clothing [1,2,3,4]. These sensors are often capable of bending or stretching while exhibiting their electromechanical properties. For example, He et al. [5] propose a facile capacitive pressure sensor optimized by a low-cost nylon netting, showing a good response sensitivity (0.33 kPa^–1^) for monitoring the pulses and clicks. This structure achieves operational stability after 1000 cycles and a response time of 20 ms. The group of Kim [6] presents hand-drawing pressure sensors using carbon nanotubes (CNTs), wet tissues, and pyralux films. The sensors are flexible, thin, water-resistant, and high-performance. With a sensitivity of 0.2 kPa^−1^, these structures are suitable for touch detection when controlling a mobile phone or tablet. In another work, Zhang et al. [7] show a capacitive pressure sensor that is capable of making measurements at low pressure (0.2 g soybean). This research uses an elastic metalized sponge (nickel-plated polyurethane sponge) as the elastic porous electrode. The sensor is applied to the grasping robot. It is clear that the most significant benefit of soft sensors is their usability in different environments, flexibility, safety, and comfort for the wearer [8,9,10]. On the other hand, the main challenges of these sensors are signal collection and stable operation in an application [11,12,13].

In recent years, flexible sensors have been extensively studied in various fields, such as medicine [14,15], healthcare [16,17], the environment [18,19], and biology [20,21]. There are some commercial products, consisting of motion-tracking clothing [22,23], medical care devices [24,25], or health alarms [26,27]. However, these studies only approach a small part of the entire system. Considering a system, we need connections, signal acquisition, processing circuits, data analysis techniques, and other components/sensors with tasks that the flexible sensors cannot yet perform. From the above approach, the paper models a comprehensive system for human health monitoring through pressure gloves and another example of a walking signal classification/tracking system. In this model, the soft capacitive pressure sensors are based on thin spacer fabric [28,29], single-walled carbon nanotubes (SWCNTs) [30], stretchable silver paste [31], and encapsulating glue [32]. This structure can generate a 3D response from the sensor under deformation. The fabricated sensor has shown good properties in response time of 7 ms, resiliency, and durability after 10,000 loading/unloading cycles. More specifically, the sensors are considered when connecting with other system components, such as processing circuits and traditional sensors. This system can classify the human walking phases using two machine learning models, including a simple deep neural network [33] and a random forest [34]. The deep neural network is a model based on a neural network containing many hidden layers, and the random forest is a classification model based on multiple decision trees. The work will provide an overview of flexible sensors in a complete embedded system.

## 2. Materials and Methods

### 2.1. Materials

Spacer fabrics are unique fabrics comprising two separate layers connected by fiber (polyester). This 3D structure creates 3D microclimates between the layers, leading to outstanding properties such as durability, flexibility, and breathability for the spacer fabric. Two outer stretch fabrics are woven from a primary blend of synthetic polyester (polyethylene terephthalate) and spandex with a ratio of 76/24 from SNT Co., Ltd., Seoul, Republic of Korea. This method of co-weaving provides excellent elasticity for many particular applications. After surface coating the polyester/spandex fibers, these fabrics will form conductive layers that are able to change the conductivity through deformations. Surface coating ink was purchased from KH Chemicals Co., Seoul, Republic of Korea; silver paste and encapsulation glue were prepared from Dycotec Materials Ltd. (Calne, UK).

### 2.2. Fabrication Method

**Figure 1:**
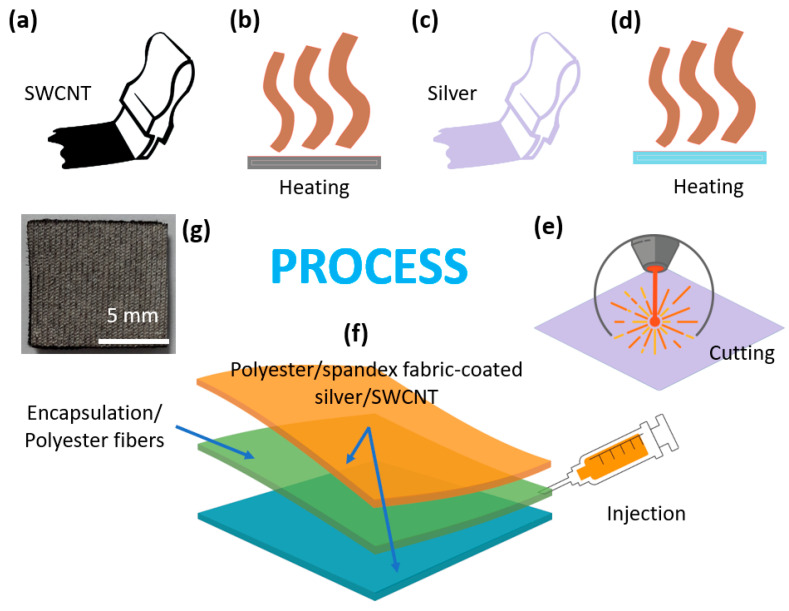
The fabrication process of a capacitive pressure sensor, including (**a**) Screen printing SWCNT, (**b**) Heating in the first, (**c**) Screen printing silver paste, (**d**) Heating in the second, (**e**) Laser cutting, (**f**) Injeting encapsulation glue, and (**g**) Real capacitive pressure sensor.

The fabrication processes of the capacitive pressure sensors are a combination of three phases: compositing the conductive layer with SWCNT and silver paste, shaping the sensors by laser cutting machines, and injecting the encapsulation glue into the spacer layer. Firstly, an ultrasonic stirring machine was used to remove the air bubble inside SWCNT ink at 0.1 wt%. The stirring machine worked for 2 h at a stirring frequency of 20 Hz, a temperature of 75 °C, and a speed of 1000 rpm. In addition, the silver paste was kept in a fridge at 4 °C in a tightly sealed bottle. Using screen-printing technology at a speed of 20 mm/s, SWCNT layers were coated on each surface of the spacer fabric (Figure 1a). The excess water inside the ink was removed with a two-way dryer (Figure 1b). This step was set up at a temperature of 200 °C, a time of 2 min, and a fan speed of 1500 rpm. Then, we applied the above process with silver paste to the two sides of the spacer fabric, as shown in Figure 1c,d. At that time, the parameters underwent a slight change. The dryer worked at a temperature of 130 °C for 15 min to remove the solvents. Secondly, the pressure sensors could achieve their own customizable shapes with a laser cutter (Figure 1e). In this step, we shaped the sensors into a square of 10 mm × 10 mm. Thirdly, the encapsulation glue was inserted inside the spacer layer by a small injection needle, as described in Figure 1f. The dryer worked at a temperature of 130 °C for 10 min to fix these glue positions. Finally, the obtained sensor is shown in Figure 1g, consisting of two electrode layers of the polyester/spandex fabrics coated with SWCNT/silver. These fabric layers achieve high conductivity, stretchability, flexibility, and quick response/recovery under pressure deformations for practical applications. Between two electrode layers, a dielectric layer was formed by encapsulation glue and polyester fibers. This structure guarantees that the sensors have strong and constant elasticity even under large forces. In addition, the thin spacer fabric would fit many small spaces for different wearable devices.

## 3. Results and Discussion

**Figure 2:**
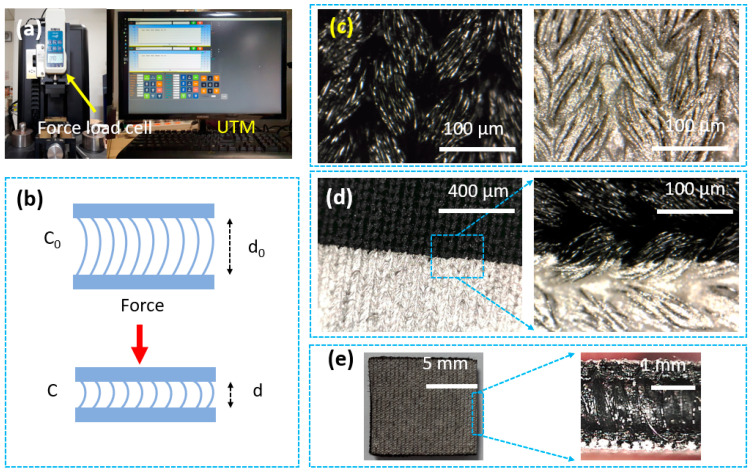
(**a**) Universal testing machine (UTM); (**b**) Working principle; (**c**) Scanning electron microscope (SEM) figures of the sensor at 100 μm; (**d**) Scanning electron microscope (SEM) figures of the sensor at the edge printing; (**e**) The final sensor and the cross view.

The sensor’s characteristics are checked by a universal testing machine (UTM), as shown in Figure 2a. Figure 2b describes the working principle of the fabricated capacitive pressure sensor. Accordingly, the dielectric layers from encapsulation and polyester fibers are compressed when loading a force. The distance between the two electrode layers (silver/SWCNT) becomes closer, which changes the capacitance of the sensor. One challenge with soft capacitive pressure is resiliency and noise under deformations. Here, the polyester filaments help ensure resilience quickly. The dielectric layer ensures minimal interference in flexible applications.

Capacitance change is calculated according to Equation (1), where *C* represents the capacitance at the compressed force, $\varepsilon_{0}$ represents the constant for the dielectric permittivity of vacuum, $\varepsilon_{r}$ represents the permittivity of the dielectric layer, *A* represents the area of the electrode layer, and $d_{0}$ represents the distance between two electrode layers, respectively. The sensitivity (*S*) can be defined from Equation (2), where $C_{0}$ is the initial capacitance, Δ*C* is the capacitance change, and P is the loading pressure.

(1) $$C_{sensor}=\varepsilon_{0}\varepsilon_{r}\frac{A}{d_{0}}$$

(2) $$S=\frac{\Delta C/C}{C_{0}}$$

From Equation (1), the capacitance change can be raised by decreasing the distance *d* and increasing the dielectric constant *ε*. This *ε* of the dielectric layer will be defined in Equation (3), where $V_{air}$ represents the volume of the air, $V_{PET}$ is the volume of the PET fibers, $V_{encapsulation}$ represents the volume of the encapsulation glue, $\varepsilon_{air}$ represents the permittivity of the air, $\varepsilon_{PET}$ is the permittivity of the PET fibers, and $\varepsilon_{encapsulation}$ is the permittivity of the encapsulation glue. It is demonstrated that the decrease in the volume of air gaps contributes significantly to the increase in capacitance obtained when compressing and releasing the force.

(3) $$\varepsilon_{r}=\left(\%V_{air}.\varepsilon_{air}+\%V_{PET}.\varepsilon_{PET}+\%V_{encapsulation}.\varepsilon_{encapsulation}\right)$$

Scanning electron microscopy (SEM) was used to analyze the surface morphology of the sensor. Figure 2c–e shows the knitted structure of two electrode layers of polyester/spandex fabrics (before and after the screen printing process). This structure consists of interconnected loops that create extremely high stretchability. The diameter of the single fibers is about 10 µm, and there are many gaps between the fibers or bundles. After the printing processes, the filaments are coated with silver/SWCNT, covering about 90% of the printed area. Figure 2c,d shows that the surface is illustrated at different steps in the overall process. Encapsulation glue is inserted with an injection needle between the gaps inside the spacer fabric. As shown in Figure 2e, the air volume is minimized to increase the dielectric constant.

**Figure 3:**
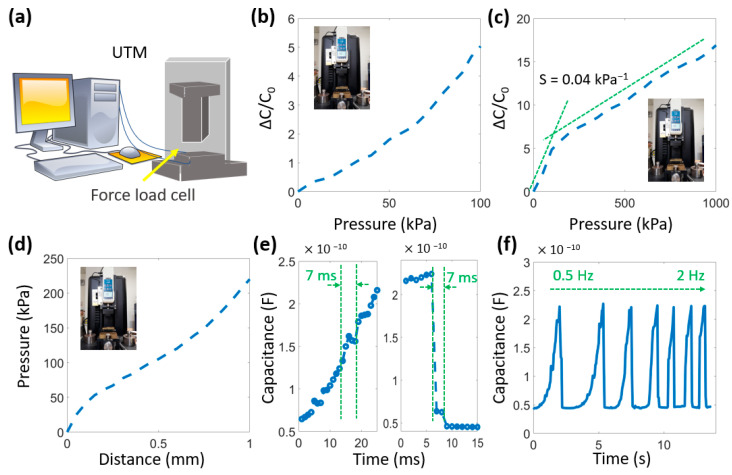
Properties of the sensor, consisting of (**a**) Diagram of UTM machine; (**b**) Capacitance change under pressure from 0–100 kPa; (**c**) Capacitance change under pressure from 0–1000 kPa; (**d**) Distance change under pressure; (**e**) Response and recovery time; (**f**) Capacitance change at different frequencies.

Figure 3 describes the capacitance change at the different levels of compression. The pressure sensitivity is about 4 × 10^−2^ kPa^−1^. Thanks to the construction of the spacer fabric, the sensor can withstand pressures of up to 1000 kPa, as seen in Figure 3b–d. It demonstrates that the sensor has an extensive working range and is suitable for many applications. The resilience of the sensor package depends mainly on the viscosity of the polyester pile fibers and the encapsulation glue. Resilience is vitally essential for practical applications that require a rapid response. Here, the response and recovery times can be seen in Figure 3e, with 7 ms at 100 kPa pressure. The main reason for this good value is the ability of the polyester fibers to release two parallel electrodes quickly. Additionally, we consider the dynamic performance of the sensor in Figure 3f. The result shows that the sensor has a stable response at different frequencies, from 0.1 to 2 Hz.

**Figure 4:**
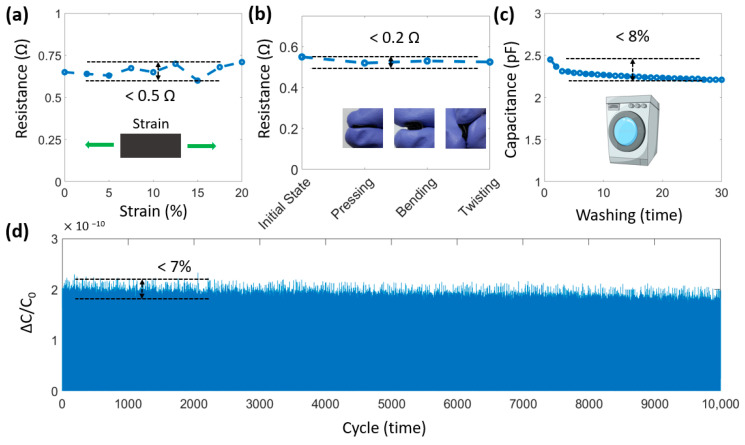
Properties of the sensor, consisting of (**a**) Resistance change under strain from 0–20%; (**b**) Resistance change when pressing, bending, and twisting; (**c**) Capacitance change after washing; and (**d**) Capacitance change under 10,000 loading/unloading cycles.

In the event of undesirable deformations, the electrode layers can still maintain high conductivity, as demonstrated in Figure 4a,b. The resistance change is under 0.5 Ω after stretching, pressing, bending, or twisting. This is due to the high stretchability of the stretchable silver paste layer. Another highlight is the ability to maintain capacitance after washing. As shown in Figure 4c, the maximum capacitance of the sensor has a slight change of 8%. Figure 4d describes the testing results to evaluate the durability of the sensor under many working cycles. Accordingly, our sensor exhibits a stable electrical function and excellent mechanical integrity (<7% change) at 100 kPa after 10,000 loading/releasing cycles. This variation in dynamic durability is mainly due to fatigue and plastic deformation of the dielectric layer (encapsulation and polyester fibers), causing permanent structural deformation. These above characteristics are significant for practical wearable systems. In addition, Table 1 summarizes some studies on flexible sensors, including their principles, thickness, sensitivity, and response time [32,33,34,35,36,37,38]. Our sensor clearly demonstrates good capabilities, such as a fast response time and small thickness, for a wide range of wearable applications.

## 4. Recognizing Walking Phases and Wearable Embedded System

**Figure 5:**
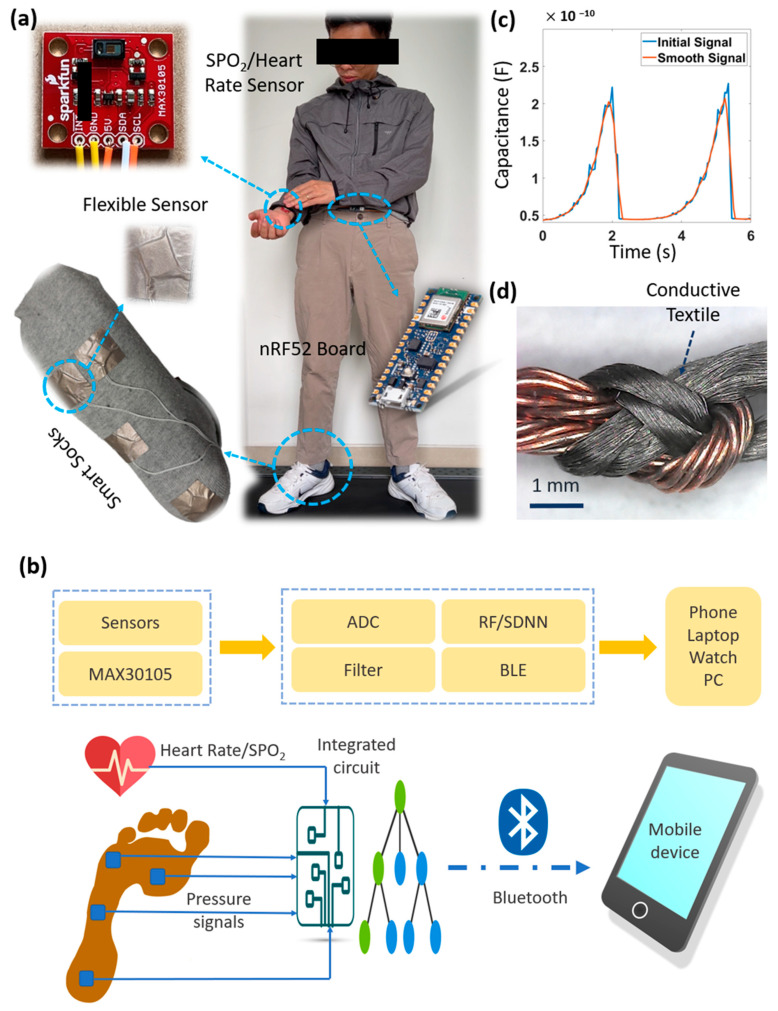
(**a**) Structure of the wearable monitoring system, including four capacitive pressure sensors (smart socks), MAX30105, and Arduino Nano 33 BLE Sense; (**b**) Working diagram of the smart wearable system; (**c**) Smooth signal; (**d**) Connection (sheet bend knot) between copper wire and conductive textile wire.

To demonstrate the ability of the fabricated sensors and wearable systems based on flexible sensors, we propose a method to monitor the overall indicators of human exercise (Figure 5a). This system will track signals such as phase, heart rate, and ${\mathrm{SPO}}_{2}\;$ratio during different walking phases. These indicators are significant for applications to analyze daily clinical health after exercise or for patients recovering from injury. To achieve this aim, the system will consist of three main parts: the smart sock-added four flexible pressure sensors, the MAX30105 module, and the Arduino Nano 33 BLE Sense module. The MAX30105 board contains two LEDs that are used to detect pulse oximetry and heart rate signals. The Arduino Nano 33 BLE Sense is a completely tiny, embedded board with many sensors that can run a small AI model. Some machine learning models are built to classify walking phases from the received signals, such as simple deep neural networks and random forests. Human walking activity can be divided into four main phases, including heel-strike (HS), hell-off (HO), toe-off (TO), and mid-swing (MS). The amplitude change of the signals is the lowest in the MS phase and the highest in the HS phase. As described in Figure 5b,c, we collect signals at a rate of 1000 samples/min from the wearable system. The analog signals are then converted to digital signals and filtered to remove noise or unusual vibrations. Some features extracted from the walking samples are applied to machine learning models. Finally, the classified phases are sent via Bluetooth to phones, tablets, or smartwatches.

Deep learning is a neural network with many hidden layers between the input and output layers. These neural networks attempt to simulate the behavior of the human brain, allowing it to learn and observe exciting patterns between data patterns. Deep learning appears in many AI applications and services, which help collect data and improve analysis without human intervention. However, the data required for a deep learning model must be large enough and take a long time to train. Due to the characteristics of the existing dataset, we chose a simple deep neural network (SDNN) model with three hidden and fully connected layers. Each layer consists of multiple nodes (neurons) and builds upon the previous layer to refine and optimize the classifier. The output of one node will become the input of the next node. Data will be passed from one layer to the next (a feedforward neural network). The total number is about 35 nodes (neurons) in hidden layers. We also evaluated the system with a random forest (RF) model. Random forest is a type of supervised machine learning algorithm for classification and regression problems. This algorithm combines the output of multiple decision trees to reach a single result. RF will build many decision trees, and each tree is unique. The prediction results are based on the aggregation of the decision trees. The RF algorithm has proven particularly powerful for tasks involving the data of soft sensors. The paper will show an overview of the capabilities of two SDNN and RF algorithms with a system that integrates flexible pressure sensors.

We evaluated the three main problems of flexible sensors when placed in an overall system without considering the complex manufacturing process. Firstly, there is a connection between the sensors and the electrical wires. The soft sensors are not created from heat-resistance materials, so soldering the metal wires directly onto the electrode layers is not reasonable. Here, the best practice is to secure part of the wire with strong adhesive and fix the rest of the wire ends with a laminating layer, as shown in Figure 5. Secondly, there is a difference in working phase between the soft sensors and other sensors in the system. These sensors can be other soft sensors or traditional sensors. The reason could be due to the flexibility of the new sensors or loose connections. Some studies used conductive fabric-type electrical wires, which further increase the possibility of noise causing instability in the received signal. The connection between the metal wire and the wire based on fabric is also an issue. We suggest twisting the two ends of the wire together, such as in a sheet bend knot, and fixing them with instant glue (Figure 5d). Then, the connected position is wrapped by a heat shrink tube. This method ensures the signal’s transmission capacity and protects the connection. A new AI model will require a lot of experimentation and tweaking in the system and signal processing method to get the best combination. However, wearables have the issue that the average deep learning or machine learning model is too large to be applicable on a small embedded board. Therefore, choosing a suitable model for each application is extremely necessary.

**Figure 6:**
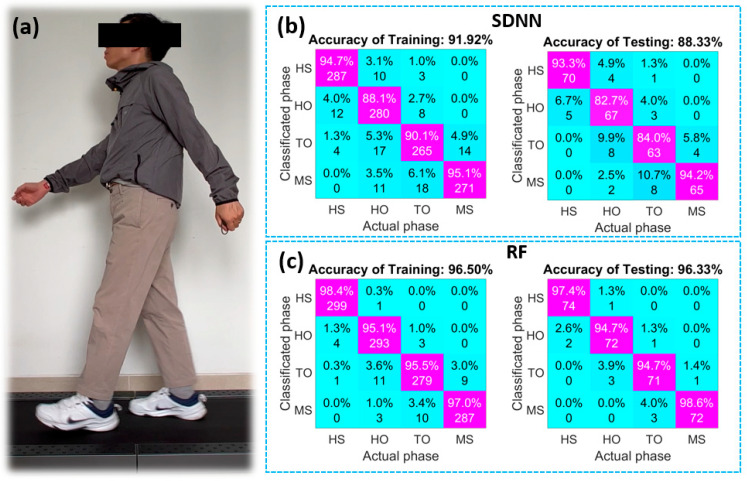
(**a**) Human walking exercise; (**b**) Confusion matrixes of simple deep neural networks; (**c**) Confusion matrixes of random forests.

Figure 6a describes the walking motion with the wearable system, including four flexible sensors and the MAX30105 module. Signals from the system are recorded with 1500 data samples, and each phase of walking motion will have 375 samples, 80% for training and 20% for testing. The performance of the models will be evaluated through the confusion matrixes, as shown in Figure 6b,c. The confusion matrix calculates the model’s metrics, such as accuracy and recall. Each column of the matrix represents the actual walking phases. In contrast, each row of the matrix represents the predicted walking phases. Each cell measures how many times a phase was correctly and incorrectly classified. High values on the diagonal line show the performance of the model. After the training process, the average accuracy reaches 91.92% for SDNN and 96.5% for RF. However, the average accuracy of SDNN is only 88.33% when testing new data. This index of RF is much better, at 96.33%. The accuracy of each phase (HS, HO, TO, and MS) is 97.4%, 94.7%, 94.7%, and 98.6%, respectively. We observe that the HS and MS phases are the easiest to distinguish, while the HO and TO phases are easily confused with each other. It is clear that the RF model is suitable for the existing dataset. Among them, the highest accuracy is obtained when classifying the MS phase (98%), and the lowest accuracy is obtained when recognizing the HO phase (94%). The practical application has demonstrated that the built-in flexible capacitive pressure sensor has a high potential for wearable devices in tracking and recognizing body movements. However, the sensor still has a limitation. There is a difference in the working ability of each position on the socks. This problem leads to asynchronous signals. At that time, the system could not comprehensively evaluate the flexible sensors. We recommend several different structures of the spacer fabric for different positions. This direction will be carried out in the subsequent studies.

## 5. Conclusions

This paper proposes a method to fabricate a flexible capacitive pressure sensor based on spacer fabric, polyester/spandex fabric, encapsulation glue, SWCNT, and silver paste. Thanks to the unique 3D structure of the spacer fabric containing polyester piles, the sensor achieves good sensitivity and extreme resilience for a variety of applications. In addition, the stretchability of polyester/spandex fabric and conductive silver paste has increased the durability of the sensor when subjected to unwanted deformations. The combination of two printing layers (SWCNT/silver) ensures the high conductivity of the electrodes. More importantly, we evaluated the capability of a practical system with various components, such as embedded boards, other soft sensors, or traditional sensors. Among the challenges a system needs to address, the connections (between the flexible sensors and the electrical wires) and the signal processing model (which analyzes the resulting data) are two problems that need to be considered in detail. On testing with two machine learning models, RF gave better results and will be used for further studies. The combination of the soft sensing system and machine learning will become a universal platform for various wearable applications in the future.

## Figures and Tables

**Table 1:** A comparison of some flexible pressure sensors.

Ref.	Principle	Thickness	Response Time	Sensitivity
[5]	Capacitive	2.698 mm	20 ms	0.33 kPa^−1^
[7]	Capacitive	>4 mm	>50 ms	-
[35]	Capacitive	1.185 mm	-	0.283 kPa^−1^
[36]	Capacitive	1.96 mm	7 ms	0.0121 kPa^−1^
[6]	Resistive	0.26 mm	70 ms	0.2 kPa^−1^
[37]	Resistive	3 mm	-	0.05 kPa^−1^
[38]	Resistive	0.15 mm	-	0.001 kPa^−1^
Ours	Capacitive	1.85 mm	7 ms	0.04 kPa^−1^

## Data Availability

The datasets generated and/or analyzed during the current study are available from the corresponding author on reasonable request.
